# Synergistic Solvent Extraction of Lanthanoids with Traditional Ligands (4-Acylpyrazolone and Bidentate Nitrogen Bases) in a Nontraditional Diluent Confirmed by Slope Analysis and NMR

**DOI:** 10.3390/molecules30040786

**Published:** 2025-02-08

**Authors:** Maria Atanassova, Nina Todorova, Svetlana Simova

**Affiliations:** 1 Department of General and Inorganic Chemistry, University of Chemical Technologies and Metallurgy, 8 Kliment Okhridski Blvd., 1756 Sofia, Bulgaria; nina.todorova@uctm.edu; 2 Institute of Organic Chemistry with Centre of Phytochemistry, Bulgarian Academy of Sciences, Acad. G. Bonchev Street, Block 9, 1113 Sofia, Bulgaria; svetlana.simova@orgchm.bas.bg

**Keywords:** lanthanoids, synergistic extraction, 4-benzoyl-3-methyl-1-phenyl-2-pyrazolin-5-one, 1,10-phenanthroline, 2,2′-bipyridine, NMR, separation factors

## Abstract

The synergistic solvent extraction of La(III), Eu(III) and Lu(III) with a chelating extractant, 4-benzoyl-3-methyl-1-phenyl-2-pyrazolin-5-one (HL), and neutral bidentate heterocyclic amines, such as 1,10-phenanthroline (S1 (phen)) or 2,2′-bipyridine (S2 (bipy)) in an ionic liquid of the imidazolium family [C_1_C_4_im^+^][Tf_2_N^−^] was investigated. Synergistic effects have been observed to result from the formation of a ternary complex in the organic phase, particularly in cases where the ligand S is a neutral synergistic agent. Examples include La(L)_2_(S2)_2_, Eu(L)_3_(S2) and Lu(L)*_x_*(S2)_2_, as well as La(L)_3_(S1)_2_, Eu(L)_2_(S1) and Lu(L)_3_(S1)*_x_*). The parameters of the solvent extraction process were determined and the influence of the synergistic agent on the extraction process was discussed. Additionally, the synergistic increase and separation factors were determined. The equilibrated organic phases were analyzed using ^1^H NMR spectroscopy to elucidate the synergism in an extraction mechanism. The role of the ionic diluent in complexation processes and selectivity was investigated with the employment of the two synergistic agents for various metal s-, p-, d- and f-cations in the periodic table, with almost 22 metal ions.

## 1. Introduction

The phenomenon observed in the field of solvent extraction chemistry, whereby the combined use of two extractants results in the extraction of a metal ion species with a significantly enhanced efficiency compared to the additive effect observed when they are used separately, is referred to as a synergism [1,2,3,4]. The well-known synergistic effect in solvent extraction chemistry, which has been extensively studied over the past six decades in various molecular diluents, involves the enhancement of the extraction of a metal ion with an acidic chelating agent, typically through the addition of a neutral ligand [5]. In comparison with these mixtures, there is a relatively small number of studies dedicated to the solvent extraction of metals using mixtures comprising a neutral bidentate ligand, particularly in an ionic liquid medium [5]. For example, the formation of synergistic adducts containing a β-diketone as the acidic component (acetylacetone, benzoylacetone [6,7,8], pivaloyltrifluoroacetone [9], thenoyltrifluoroacetone [10,11,12,13,14,15,16], LIX 54 [17] and 1-phenyl-3-methyl-4-benzoyl-5-pyrazolone [18]) and bidentate chelating agents, 1,10-phenanthroline or 2,2′-bipyridyl, have been documented for a multitude of metals under a variety of experimental conditions in traditional organic diluents. It is noteworthy that Kassierer and Kertes [10] achieved a remarkably high synergistic effect, up to 10^6^, through the combination of two bidentate chelating agents: the β-diketone and a nitrogen base. The two molecules, 1,10-phenanthroline and 2,2′-bipyridine, are among the most well-known base polypyridyl units that exhibit a high complexation affinity toward many metal ions as they represent versatile starting blocks for molecular recognition in supramolecular chemistry [19,20,21].

One significant area of investigation currently undergoing intensive research is the potential replacement of traditional volatile, toxic and/or flammable organic diluents, which raises safety and environmental issues nowadays with ionic liquid compounds (ILs) [22,23,24,25]. In the field of metal solvent extraction, separation and recycling by liquid–liquid processes, hydrophobic ILs of the imidazolium type [C_n_C_m_im^+^][Tf_2_N^−^] are preferred one, avoiding the problems linked to high viscosity. This type of compounds has already demonstrated their efficacy as organic media for a wide range of metals in the periodic table, including lanthanoids [5]. Cations are denoted as [C_n_C_m_im^+^] for *n*-alkyl-*m*-alkylimidazolium, while the anions [Tf_2_N^−^] correspond to (CF_3_SO_2_)_2_N^−^ (bis(trifluoromethylsulfonyl)imide) [26,27].

Indeed, most rare earth elements are employed in a multitude of sectors, both traditional and modern, due to their diverse and advantageous characteristics (such as electronic, luminescent, magnetic and catalytic properties). Nevertheless, given the resemblance in the chemical and physical characteristics, the intra-group separation, especially neighboring elements, is a complex technological challenge that warrants substantial research effort [27]. On the other hand, lanthanoids are applied as critical metals with a strategic role in modern industries, making their effective recovery from various secondary sources through recycling a highly valuable sustainable technology to secure the Earth’s green future. Moreover, modern green technology requires non-renewable raw materials sourced from primary geological resources (mines) or secondary supply (reuse or recycling as much as possible) but the research ambition is to achieve a fully circular economy un depending on the type of applied chemical technology [1,2,3]. In general, liquid–liquid extraction is a well-established hydrometallurgical process for the separation of metals.

This fundamental study concerns the solvent extraction of three trivalent ions, namely La^3^⁺, Eu^3^⁺ and Lu^3^⁺, which have been selected as representatives of the beginning, middle and end of the 4f series, respectively. The extraction process was conducted using the water-immiscible imidazolium-type ionic liquid [C_1_C_4_im^+^][Tf_2_N^−^], which was employed as a diluent for the widely used anionic ligand from the 4-acylpyrazolone family as a chelating extractant, both when used alone and in combination with bidentate nitrogen compounds such as 1,10-phenanthroline or 2,2′-bipyridine. The primary objective is to ascertain the extent of the synergistic enhancement and selectivity produced by the studied mixtures. However, the data interpretation and the slope analysis results assessment may be insufficient from the perspective of solvent extraction mechanism research in an ionic diluent. Nevertheless, they provide sufficient information about the utility of the employed solvent systems in prospective applications. The principal focus is, of course, the capacity to achieve effective selectivity in the intra-group 4f series through the utilization of diverse solvent systems. Finally, organic phases of biphasic solvent systems with the required ligand concentrations, consisting mainly of the [C_1_C_4_im^+^][Tf_2_N^−^] diluent after contact with the corresponding aqueous phase, were also studied for the first time by ^1^H 1D NMR without any dilution.

## 2. Results and Discussion

### 2.1. Solvent Extraction of La(III), Eu(III) and Lu(III) Ions with HL Alone and Two Synergistic Mixtures HL–1,10-Phen or HL–2,2′-Bipy

**Figure 1:**
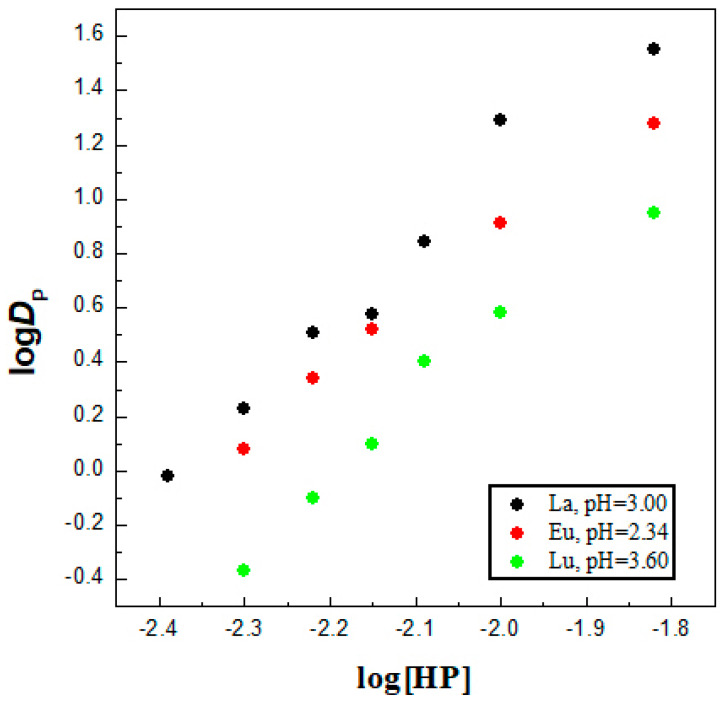
Log D vs. log [HL]_IL_ plots at constant pH for La^3+^ (slope ~2.89), Eu^3+^ (slope ~2.50) and Lu^3+^ (slope ~2.76) in [C_1_C_4_im^+^][Tf_2_N^−^]. The experiment was separately performed under the presence of a single metal ion in each aqueous phase.

Firstly, the solvent extraction performance of the chelating agent HL alone diluted in an ionic diluent was assessed towards La^3+^, Eu^3+^ and Lu^3+^ ions as representatives of light, middle and heavy 4f series, *ceteris paribus*. In fact, neutral extractants are readily soluble in imidazolium-based ILs, unfortunately acidic extractants sometimes showed poor solubility and this greatly affects their extraction behavior. The stoichiometric information about the extraction processes, called “slope analysis” method, is based on an examination of the variation in D (the distribution ratio) as a function of the relevant experimental variables [5]. In the IL system, slope analysis of Ln solvent extraction with HL molecule used alone was investigated as a function of ligand concentration in the organic oil phase at a constant pH of the aqueous phase (Figure 1) in order to determine the reaction mechanism, as well as the stoichiometry, of the Ln^3+^–HL complexes formed in the [C_1_C_4_im^+^][Tf_2_N^−^] phase. As shown in Figure 1, the obtained plots for HL concentration were linear with a slope very close to three for solvent extraction of La^3+^, Eu^3+^ and Lu^3+^ ions, suggesting that three ligand molecules were required to extract one Ln^3+^ ion into the IL, in that a 1:3 metal/ligand ratio complex was formed. The formation of 4f metal chelates, LnL_3_, with 4-acylpyrazolones in an IL media was also noted previously [28]. The formation of self-adducts LnL_3_·HL when acylpyrazolones are used as chelating extractant in typical molecular diluent was found in some other studies [29,30], but in refs. [31,32], the extraction of the LnL_3_ chelate complex was also reported. However, in an ionic liquid media, the situation is much more complicated as various metallic species have been usually established due to the ionic character of the diluent [5]. In general, the solvent extraction mechanism, when the metal cation forms a chelate complex with available ligands and moves into an organic phase as a single, neutral uncharged species, was represented by the following equation [28]:

Ln^3+^_aq_ + 3HL_org_ ⇌ LnL_3,org_ + 3H^+^_aq_ (1)

where subscripts “aq” and “org” denote the aqueous and organic phases, respectively.

The coordination sphere of the trivalent ion is unsaturated in this six-coordinate chelate complex, so the Ln(III) ion can expand it by forming an adduct formation. Depending on the experimental protocol, with participation of water molecules, for example LnL_3_·(H_2_O)*_x_*, *x* = 2 or 3 [5]. Therefore, in an IL solution, the completion of the coordination sphere can be achieved either through binding with the Tf_2_N^−^ anions or by water molecules. The last ones are known to transfer to the IL phase, ca. 12,000 ppm under such chemical conditions [5]. In fact, Tf_2_N^−^ is one of the weakest coordinating anions, so does not compete favorably with H_2_O molecules for the Ln(III) coordination sphere [5].

**Figure 2:**
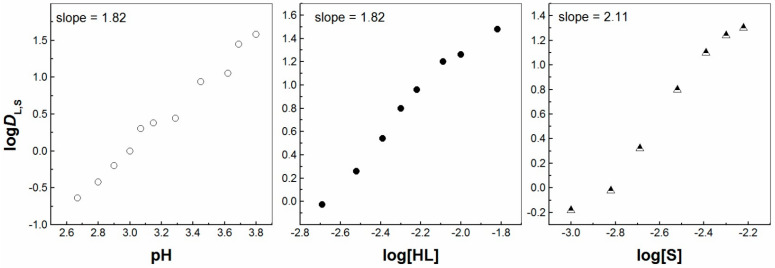
Log *D*_L,S_ vs. log pH ([HL] = 5 × 10^−3^ mol/dm^3^ and [S] = 3 × 10^−3^ mol/dm^3^), log *D*_L,S_ vs. log[HL]_IL_ ([S] = 3 × 10^−3^ mol/dm^3^ at pH = 3) and log *D*_L,S_ vs. log[S] ([HL] = 5 × 10^−3^ mol/dm^3^ at pH = 2.78) plots for solvent extraction of La^3+^ with the HL–bipy mixture in [C_1_C_4_im^+^][Tf_2_N^−^].

**Figure 3:**
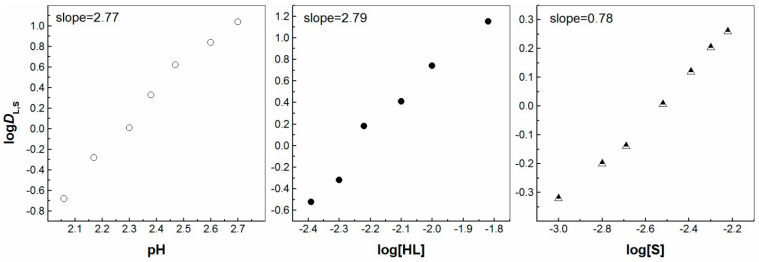
Log *D*_L,S_ vs. log pH ([HL] = 5 × 10^−3^ mol/dm^3^ and [S] = 3 × 10^−3^ mol/dm^3^), log *D*_L,S_ vs. log[HL]_IL_ ([S] = 3 × 10^−3^ mol/dm^3^ at pH = 2.30) and log *D*_L,S_ vs. log[S] ([HL] = 5 × 10^−3^ mol/dm^3^ at pH = 2.23) plots for solvent extraction of Eu^3+^ with the HL–bipy mixture in [C_1_C_4_im^+^][Tf_2_N^−^].

**Figure 4:**
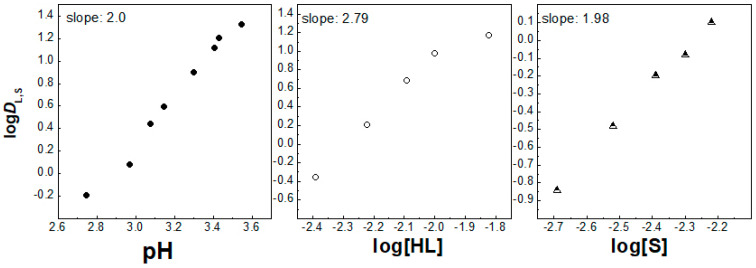
Log *D*_L,S_ vs. log pH ([HL] = 5 × 10^−3^ mol/dm^3^ and [S] = 3 × 10^−3^ mol/dm^3^), log *D*_L,S_ vs. log[HL]_IL_ ([S] = 3 × 10^−3^ mol/dm^3^ at pH = 2.55) and log *D*_L,S_ vs. log[S] ([HL] = 5 × 10^−3^ mol/dm^3^ at pH = 2.55) plots for solvent extraction of La^3+^ with the HL–phen mixture in [C_1_C_4_im^+^][Tf_2_N^−^].

**Figure 5:**
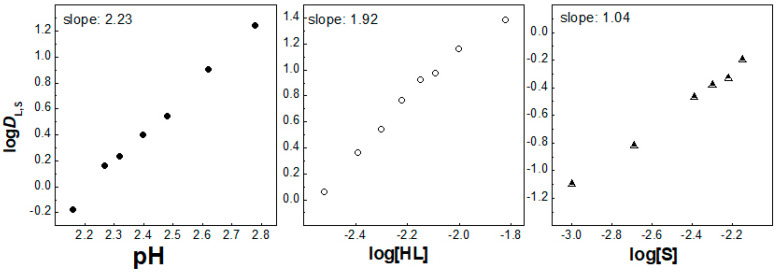
Log *D*_L,S_ vs. log pH ([HL] = 5 × 10^−3^ mol/dm^3^ and [S] = 3 × 10^−3^ mol/dm^3^), log *D*_L,S_ vs. log[HL]_IL_ ([S] = 3 × 10^−3^ mol/dm^3^ at pH = 2.25) and log *D*_L,S_ vs. log[S] ([HL] = 5 × 10^−3^ mol/dm^3^ at pH = 2.25) plots for solvent extraction of Eu^3+^ with the HL–phen mixture in [C_1_C_4_im^+^][Tf_2_N^−^].

Secondly, the liquid–liquid extraction of La^3+^, Eu^3+^ and Lu^3+^ ions was carried out by two molecules forming the acidic/neutral couple in order to produce the synergistic enhancement in an IL media, i.e., [C_1_C_4_im^+^][Tf_2_N^−^]. The corresponding plots of log*D*_I,S_ vs. pH; log*D*_I,S_ vs. log [HL] and log*D*_I,S_ vs. log[S] were determined by the investigations of the synergistic extraction of three lanthanoid ions. These plots are shown in Figure 2, Figure 3, Figure 4, Figure 5, Figures S1 and S2 (Supplementary Materials). The obtained values of the *D* ratios of the mixed synergistic systems are greater than those of individual extractants [33,34]. This observation clearly reveals the positive synergistic extraction effects under the applied experimental conditions herein. In other words, the synergistic enhancement in an IL media is realized, but it is difficult to establish the nature of the exact extracting Ln^3+^ complex formed in this oil phase as a different stoichiometry is revealed for the three 4f ions. The arrangement of one unique synergistic complex in the organic phase is probably an unfavorable extraction mechanism applying one solvent system. Thus, the organic phase synergistic reaction could not be expressed by one equation only. This is due to the intensive ion exchange between the two liquid phases, including the loss of the IL cation or anion to the aqueous phase, in contrast to molecular diluents [5]. Therefore, in the presence of the second extractant, 2,2′-bipyridine molecules (S2), the La^3+^ and Eu^3+^ synergistic extraction process can be expressed by the equations:

La^3+^_(aq)_ + 2HL_IL_ + 2S2_IL_ ⇌ La(L)_2_(S2)_2IL_ + 2H^+^_(aq)_, (2)

with the participation at least two chelating ligands and two molecules of the synergistic agent.

The lanthanoid mononuclear β-dicarbonyl complexes generally have very high coordination numbers (C.N.), i.e., varying from 6 to 12, with 8 and 9 being rather common. This phenomenon depends on the ionic radii of the investigated ions (La^3+^, Gd^3+^ and Lu^3+^: 103.2, 93.8 and 86.1 pm, respectively), the employed ligand, the reaction medium, the diluent in use and experimental conditions, such as the temperature or ligand to metal ratio [35]. Generally, when ILs are employed, the solvent extraction ability of a neutral ligand is observed to be almost equal to that of the principle acidic compound, even at low concentrations [5].

Eu^3+^_(aq)_ + 3HL_IL_ + S2_IL_ ⇌ Eu(L)_3_(S2)_IL_ + 3H^+^_(aq)_, (3)

with the participation of three chelating ligands and one synergistic agent.

The proposed complex is, at minimum, eight-coordinated as well, from which it follows that the arrangement of the europium complex in the organic ionic medium follows the generally accepted rules in solvent extraction chemistry. Many years ago, the four conditions for the synergistic extraction of a metal with a mixture including acidic/neutral ligands in molecular diluents were indicated, leading to a considerable effect [36], i.e., formation of a metal chelate complex. The second extractant, i.e., the synergist, can displace any coordinated water from a chelate, rendering it less hydrophobic. On the other hand, the second extracting compound is less coordinated than the first one. Moreover, the maximum coordination number achieved by metal, as well as the obtained architecture of the complex, should be favorable. In synergistic systems, the chelating compound (HL) typically deprotonates to form an anionic ligand L^−^ that can chelate with the metal ion, M*^n^*^+^. At the same time, the neutral ligand, or in other words, the synergistic agent S, replaces any remaining water molecules from the coordination shell of the neutral complex (chelate), enhancing its solubility in the organic phase. Generally, this process can be expressed as:

M*^n^*^+^ + *n*HL + *x*S ⇌ ML*_n_*·S*_x_* + *n*H^+^ (4)

Unfortunately, it is difficult to establish the exact nature of the formed complex for the last cation from 4f series with HL–bipy mixture in [C_1_C_4_im^+^][Tf_2_N^−^] media, as only the number of S2 molecules could be derived approximately from the obtained results (Figure S1): Lu(L)*_x_*(S2)_2_.

On the other hand, the number of coordinated synergistic molecules was preserved when 1,10-phenantroline was introduced to the solvent system for La^3+^ and Eu^3+^ ions, when again two and one molecules participated in the extracted complexes, respectively (Figure 4 and Figure 5). Despite that, the picture changes with the chelating extractant, probably due to steric hindrances related to the size of the target 4f ion. As a result, in the presence of the second extractant, 1,10-phenatroline (S1), the synergistic extraction of both 4f ions can be expressed by the equations:

La^3+^_(aq)_ + 3HL_IL_ + 2S1_IL_ ⇌ La(L)_3_(S1)_2IL_ + 3H^+^_(aq)_, (5)

Eu^3+^_(aq)_ + 2HL_IL_ + S1_IL_ ⇌ [Eu(L)_2_(S1)]^+^_IL_ + 2H^+^_(aq)_, (6)

Lu^3+^_(aq)_ + 3HL_IL_ + *n*S1_IL_ ⇌ Lu(L)_3_(S1)*_n_*_IL_ + 3H^+^_(aq)_, (7)

The results show that under the experimental conditions, Lu^3+^ ions are extracted as neutral adduct complexes. However, the stoichiometry of the extracted species in the organic IL phase depends on the pH, Ln^3+^, HL or S1 concentrations, the solubility of ILs’ components in the aqueous phase, etc. In other words, the number of coordinated S1 molecules is unclear for this heavy 4f ion (Figure S2).

In terms of contemporary priorities, the solvent extraction systems must be adjusted in a way to accommodate the green chemistry, both achieving an efficient extraction process and having good selectivity as a target in order to minimize the possible contaminants. For instance, the undesirable IL’s component solubility in the aqueous phase, which, in general, has a pronounced impact on metal extraction efficiency, can be successfully reduced when bidentate nitrogen bases are employed in the solvent systems [5]. Thus, an improved understanding of solvation phenomena in both phases, aqueous and organic oil media, of the design of complexing agents that do not create environmental hazards or further waste disposal complications when their utility is accomplished (CHON principle) can be suggested for future research regarding green and sustainable chemistry. Moreover, after forward synergistic solvent extraction, the studied Ln^3+^ ions were easily stripped from the investigated organic phases. The achieving metal removal percentage is ca. 95%, using 0.8 mol/dm^3^ HCl acid as a stripping agent for the back extraction process.

The values of the synergistic coefficients calculated in the present study, as well as some previously published data, are given in Table 1. It is seen that lanthanoids are extracted synergistically using both HL–phen and HL–bipy mixtures, (logSC ≥ 0). However, the data show that the addition of bidentate nitrogen bases molecules to the chelating ligand causes a rather small synergism, i.e., the values are below one order of magnitude in ionic media. In addition, concerning the synergistic effects in [C_1_C_4_im^+^][Tf_2_N^−^] media, the two investigated solvent systems display different efficiencies across the 4f series as well. Further, the synergistic solvent extraction of five representatives of 4f series with a chelating extractant 4-benzoyl-3-phenyl-5-isoxazolone (HPBI) or 2-thenoyltrifluoroacetone (HTTA) and the two neutral bidentate heterocyclic amines S1 and S2 in CHCl_3_ was investigated nearly 14 years ago. Therefore, synergism as a phenomenon was reported to be due to formation of the ternary complex in the molecular organic phase, like Ln(PBI)_3_·S or Ln(TTA)_3_·S. The synergistic enhancement was established by Atanassova for all five 4f metals and mixtures of extractants (logSC > 0), and it changes approximately from 10^2^ to 10^6^. So, the two bidentate ligands, the Lewis base phenanthroline or bipyridyl, in combination with an acidic chelating agent, produce a very strong synergistic effect on the solvent extraction of lanthanoids in molecular diluents, like CHCl_3_ [37]. Those data also show that the SCs obtained for the mixtures including 1,10-phenantroline as a synergistic agent are approximately an order of two and one higher magnitude than that when 2,2′-bipyridyl is used in combination with the two chelating ligands, HTTA or HPBI, respectively. This observation confirms the findings of previous investigations in which the synergistic effect decreases in the order phen > bipy, with differences of up to two orders of magnitude [11]. The larger synergistic effect obtained with the 1,10-phenanthroline compound applied as a synergistic agent is probably due to its stronger donor properties [17]. A similar conclusion could be drawn to some extent for the present study, using an ionic diluent as an organic media. On the other hand, the relatively small synergistic increase observed with ILs in solvent extraction chemistry is, of course, a common finding compared to traditional organic diluents [17]. Further, we must pay attention that the values are logarithmic, i.e., significant synergism was found in addition to the positive effect of ILs in the metal extraction process.

### 2.2. Chemical Modeling of Solvent Extraction Processes by NMR Analysis

**Figure 6:**
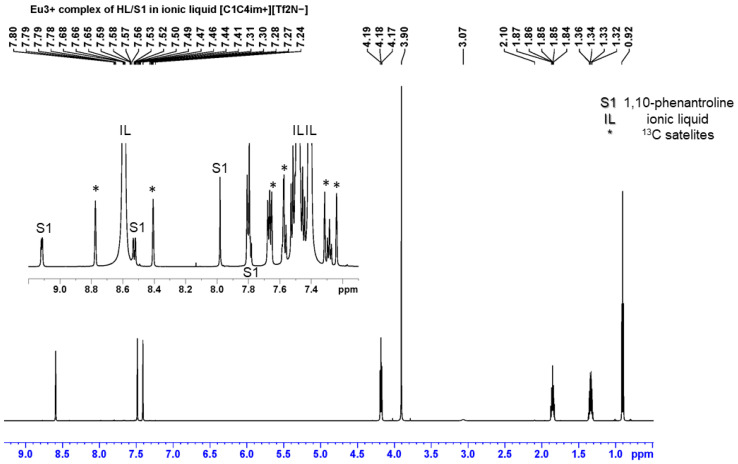
Representative ^1^H NMR spectrum of HL/S1 in [C_1_C_4_im^+^][Tf_2_N^−^], without dilution of the organic phase with deuterated diluent.

One goal of this scientific investigation is to propose a plausible solvent extraction model, as simple as possible, that quantitatively captures the reaction process correctly according to the qualitative data obtained. First, a slope analysis was performed to evaluate the stoichiometry due to the synergistic extraction of Ln^3+^ ions in an IL media. To shed new light on the synergistic phenomenon, the phase behavior of the binary system, i.e., the chelating ligand plus the synergistic agent, is studied by a detailed analysis of the ^1^H NMR spectra. For the purposes of spectroscopic interpretation, all ionic liquid phases are analyzed after phase separation of organic–aqueous layers using the Eu^3+^ cation (see Table S1 in the Supplementary Materials). To the best of our knowledge, this is the first time that this phenomenon has been detected directly in an IL without the additional application of deuterated diluents and the corresponding dilution of the organic phases used. This approach is novel in the field and extremely labor-intensive, although highly plausible. Such a scenario confirms the hypotheses formulated (as seen in Figure 6, Figures S3 and S4 and Table S1) the involvement of the second synergistic ligand in the chelating metal species during the solvent extraction process. The striking point here is the high value of uncoordinated ligands used, which are usually in excess of the metal concentration in the vicinity of the interface, and the amount of diluent which somehow predominated in the screened NMR samples.

Generally, the enhancement in the synergistic solvent extraction of metallic species may depend on the nature and strength of the possible interaction between the two ligands [38]. It should be noted that a weak but detectable interaction between the two organic extractants (HL and S1 or S2) has been detected in the spectra between them in an IL, mainly reflected in the chemical shift changes in the signals of HL. This trend is like that described for acidic–neutral ligand couples [39]. On the contrary, the metal–synergistic agent interaction with Eu^3+^ ions induces higher shift changes in the neutral bidentate heterocyclic amines (Table S1).

Actually, proton, carbon, fluorine and phosphorus NMR spectra have previously been used to investigate the possible interactions between a series of imidazolium-based ILs and several extractants, acidic chelating or neutral ligands [40]. It was shown by Kurteva et al. that no interactions occur in chloroform-D solution independently of the length of the imidazolium alkyl chain or of the structure of the ligand molecule [40]. In other words, these IL compounds could be considered as pure diluents in the solvent extraction systems.

### 2.3. Solvent Extraction, Synergism and Selectivity Across the Periodic Table

**Figure 7:**
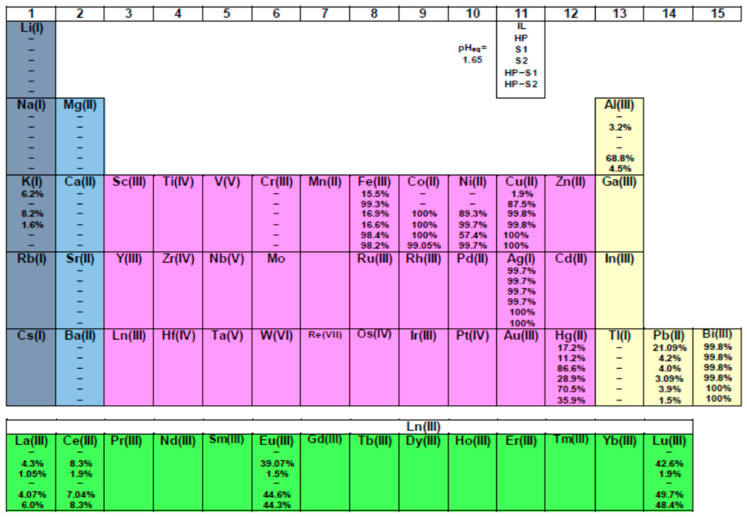
Solvent extraction performance of pure IL and HL (1 × 10^−2^ mol/dm^3^), S1 and S2 (8 × 10^−3^ mol/dm^3^) ligands diluted in [C_1_C_4_im^+^][Tf_2_N^−^] as well as the two mixtures (HL–S1 and HL–S2: 1 × 10^−2^ mol/dm^3^ − 5 × 10^−3^ mol/dm^3^) for 22 metal ions (see the explanation under no. 11 cell). The reported extractability values (%) represent the average of three measurements with deviation less than 5%.

On the whole, green technology likely requires non-renewable raw materials sourced from primary geological resources like mines or a secondary supply via reuse or recycling. Nowadays, the ambition is to create a fully circular economy, in which demand can be satisfied only by recover of almost all metals in the periodic table [39]. Unfortunately, modern society is not yet at that point, even scientifically. The competitive solvent extraction test of almost 22 metal ions by the chelating ligand HL and its mixtures has also been conducted in order to evaluate the role of a second molecule added into the solvent system, Figure 7. Therefore, the studied 22 metal ions could be classified approximately in the following two groups, according to the degree of S1 or S2 extraction performance in an IL media:

(1) Unextractable metal ions, % E < 1: Li^+^, Na^+^, Tl^+^, Ca^2+^, Mg^2+^, Ba^2+^, Sr^2+^, Al^3+^, Cr^3+^. At the same time 4f ions could not be extracted with S2 compound used alone. It can be said that the process is ineffective for K^+^ and Pb^2+^ ions.
(2) Overall extraction, % E ≥ 90: Cu^2+^, Ni^2+^, Co^2+^, Bi^3+^, Ag^+^; while Hg^2+^ only with S1. As a matter of fact, both Bi^3+^ and Ag^+^ ions could be extracted with pure IL without the need for any complexing agent. The same can be said for iron, based on the data obtained: Fe^3+^: ≤17%.

Generally, the S1 compound presents a high complexation constant with d-, and to a lesser extent, f-block cations due to their stabilization by favorable enthalpic contributions arising from the formation of strong coordination bonds with the nitrogen atoms [41]. In fact, the implications of the relatively lower affinity of M(III) ions for pyridyl donors in aqueous solution than of M(II) ions is of considerable importance in the design of ligands for selective complexation, particularly in cases where this involves the trivalent Ln(III) and Am(III) ions [42]. Furthermore, in essence, the rigidity of the 1,10-phen molecule and the permanent juxtaposition of the nitrogen donor atoms somehow provides an entropic advantage relative to its 2,2′-bipyridine analogs, resulting in enhanced kinetics of complex formation, as well as increased surface activity at the oil–water interface due to its hydrogen bonding with water molecules [43]. The following ions, Co^2+^, Ni^2+^, Li^+^ and Ln^3+^, deserve special attention as a part of green sustainable technologies. The results obtained in Figure 7 indicate that Co^2+^ and Ni^2+^ are quantitatively extracted up to 100% applying S1 or S2 ligands, contrary to alkaline and rare earth ions, which is a serious advantage in terms of separation from secondary resources as a diverse range of acceptable sources for the metals via recycling. The predominance of the S1 over the S2 molecule is only observed herein for mercury and of course in the lanthanoid series. In other words, excluding the second series of ions for the first one, we can expect a synergistic effect using the mixed solvent systems for their extraction, especially the lanthanoids. The picture obtained for cobalt and nickel, which are not extracted with the chelating acid ligand HL or an IL, is also of interest. On the other hand, Fe^3+^ is approximately 100% extracted by the HL compound, which means that there is no need for a second ligand addition. Therefore, a synergistic increase could occur for the trivalent lanthanoids and Al^3+^, given the fact that this phenomenon is not at all characteristic for metals from groups 1 and 2 of the periodic table [5,39]. And if there is insignificant synergism achieved for 4f ions (~5–8% increment), then for aluminum, the liquid–liquid extraction process is more than effective with the participation of 1,10-phenatroline as a synergistic agent.

Regarding the selectivity, it seems that metals such as Ag, Bi, Pb and Fe can be relatively easily separated from the representatives of the 4f series only with an ionic liquid without the need for additional extracting molecules in the solvent system. On the other hand, the presence of a chelating ligand affects the selectivity of metals like Fe, Cu and the light lanthanoids. For example, the calculated values of SFs of the corresponding pairs are very high: Fe/La = 3288, Fe/Ce = 1547 as well as Cu/La = 165, Cu/Ce = 77.

Unfortunately, as a common rare earth-associated impurity, usually aluminum (Al) in Ln^3+^ solution, not only affects the operation of the purification process, but also greatly reduces the final quality of products. Therefore, it is imperative to somehow develop new methods for the efficient removal of Al from Lns [44]. Hu et al. have recently reported that the SF of the Al/Gd pair could be up to 340 in [(CH_2_)_7_COOHpyr^+^][Tf_2_N^−^]/[C_1_C_4_im^+^][Tf_2_N^−^] a solvent system, as the removal rate of Al^3+^ is 97.1% and the recovery rate of Gd^3+^ is 85.6% through the one-time extraction step, which was significantly higher than the results reported in the literature at the time. The resulting separation between the two cations is too high considering that the extraction efficiency of both ions is also quite high. Despite this, the results obtained by Hu and co-workers are far away from those obtained in this study for HL and HL–S2 systems: (Eu/Al) 19.2 and 16.8, as well as (Lu/Al) 22.3 and 19.8, respectively.

Considering the low pH of the experimental conditions, it is normal to obtain a high separation in the 4f series between the light and heavy lanthanoid pairs. The most significant SFs are observed for pairs of light–medium and light–heavy pairs due to the synergistic solvent extraction process with the participation of 1,10-phenantroline, as shown in Table 2. This contrasts with the generally accepted observation that the separation becomes poorer as the extractability increases in the synergistic solvent extraction of metals. However, the investigated mixture, HL–S2 only produces better selectivity for Eu/Ce and Lu/Ce pairs in comparison with HL ligand used alone.

In accordance with what is known in molecular diluents, the comparison of selectivity data shows that the SFs are higher for the lighter lanthanoids and the Eu/La pair and extremely low for the heavy Lu/Eu pair, independently of the extracting phase nature, ionic or molecular (CHCl_3_), as shown in Table 2. However, the impact of the diluent has already been demonstrated on multiple occasions for various solvent systems in the field of extraction chemistry [45]. In other words, in comparison to CHCl_3_, the ionic liquid offers increased values of distribution ratios *D*, at the expense of a leveling off of the SFs. Despite this, the extracted metal complex would be easier to rearrange in an organic diluent with small carbon chain length in terms of the energetics of the physical distribution of metal solvate. In fact, the SF is a complex function of the chemistry of the metal ions in the two liquid phases, aqueous and organic [46]. Unfortunately, it is also worth mentioning that the ILs prices are generally expensive, and this can result in significant process needing to be justified.

It is interesting to compare the SFs obtained for the solvent extraction of lanthanoids with the same or various chelating extractants (thenoyltrifluoroacetone (HTTA), 4-benzoyl-3-phenyl-5-isoxazolone (HPBI) or 1-phenyldecane-1,3-dione, the active component in the commercial extractant LIX 54) and various synergists, viz. the crown ethers, calix[4]arenes or diphenylsulfoxide. Unfortunately, limited comparisons are possible because data are not available for all lanthanoid metals. The calculated separation factors of two pairs, Eu/La and Lu/Eu, obtained from these systems are listed in Table 3. As can be seen from the data presented in the table, better selectivity is observed with the participation of molecular systems, as compared to Table 1. On the other hand, it clearly reveals the influence of an empty (f^0^), half-filled (f^7^) and full f-subshell (f^14^) on lanthanoid’s refinement. The results somehow show the influence of Z on the loss of selectivity across the 4f series, which implies an advantage in the distinction of light, middle and heavy ions. Therefore, recycling, and the potential recovery of 4f metals from end-of-life technological objects, for example, could benefit to some extent from the absence of significantly complicated adjacent lanthanoids’ intra-group separation at these technological lines by the further application of ILs.

## 3. Materials and Methods

### 3.1. Materials

The commercial product 4-benzoyl-3-methyl-1-phenyl-2-pyrazolin-5-one (HL) with a purity higher than 99% (Fluka, Buchs, Switzerland), 2,2′-bipyridine (Merck (Darmstadt, Germany), p.a.) and 1,10-phenanthroline (Merck, p.a.) were used as supplied (the structural formulas of the extractants are presented in Figure S5). Stock solutions of the lanthanoid ions were prepared from their oxides (Fluka, puriss) by dissolving them in concentrated hydrochloric acid and diluting them with distilled water to the required volume. The diluent was 1-butyl-3-methylimidazolium-bis(trifluoromethanesulfonyl)imides, (purity, 99.5%, average water content is ca. 200 ppm) purchased from Solvionic (Toulouse, France). The Arsenazo III (Fluka, Switzerland) was of analytical purity, as were the other reagents used.

### 3.2. Extraction Procedure

The experiments were carried out using aqueous and organic phases in volumes of 2 cm^3^ and 1 cm^3^, respectively. The extractant solutions were prepared by precisely weighted samples. The samples were shaken mechanically for 2 h at room temperature (22 ± 2) °C, which proved sufficient to achieve equilibrium. The upper layer contained the aqueous phase and the bottom layer included the organic IL phase. After the separation of the phases, the metal concentration in the aqueous phase was determined spectrophotometrically (S-20 Spectrophotometer Boeco (Hamburg, Germany)) using Arsenazo III [52]. The concentration of the metal ion in the organic phase was obtained by the material balance. These concentrations were used to calculate the distribution ratio. The acidity of the aqueous phase was measured by a pH meter with an accuracy of 0.01 pH unit (211 HANNA digital pH meter (Smithfield, RI, USA)). The pH of the aqueous solutions was adjusted by the addition of either HCl or NaOH to a solution of 0.1 mol/dm^3^ 2-morpholinoethanesulfonic acid (MES) buffer. The initial concentration of the metal ions was 2.5 × 10^−4^ mol/dm^3^ in all experiments.

The distribution ratio (*D*) at equilibrium was calculated as:

(8) $$D=\frac{{\left[M^{n+}\right]}_{aq,in}-{\left[M^{n+}\right]}_{aq,f}}{{\left[M^{n+}\right]}_{aq,f}}\times \frac{V_{aq}}{V_{IL}}$$

where [M^n+^]_aq_,_in_ is the concentration of M^n+^ ion in the aqueous phase before liquid–liquid extraction tests, [M^n+^]_aq_,_f_ is the concentration of the same metal ion in the aqueous phase after extraction. In general, V_aq_ and V_IL_ are the volumes of aqueous and organic phases used to perform experiments, herein 2:1 *v*/*v* extraction. For instance, duplicate experiments showed that the reproducibility of *D* measurements was generally within 95%.

The synergistic enhancement upon the addition of a second extractant can be assessed using synergistic coefficients calculated as:

SC = log (*D*_1,2_/*D*_1_ + *D*_2_) (9)

where *D*_1,2_, *D*_1_, and *D*_2_ denote the distribution coefficient of a metal ion using mixture of extractants (*D*_1,2_) and the same extractants when used separately (*D*_1_ and *D*_2_).

For competitive extraction tests, a volume of 2 mL of the prepared aqueous solution containing various M^n+^ metal ions (the corresponding nitrate salts were used, M^n+^(NO_3_)_n_/M^n+^(NO_3_)_n_·*x*H_2_O) was equilibrated for 3 h (1500 rpm) with a 2 mL organic phase, which includes the studied ligand molecule(s). After phase separation, the metal ions concentrations in the aqueous solution were determined by ICP-OES (“Prodigy” high-dispersion ICP-OES, Teledyne Leeman Labs, Hudson, NH, USA). The extractability (% E) was evaluated according to the following formula:

(10) $$\mathrm{extractability}=\frac{\;{{[M}^{n+}]}_{aq,in}-{{[M}^{n+}]}_{aq,f}}{{{[M}^{n+}]}_{aq,in}}\times 100$$

The metal separation between studied elements herein in the periodic table can be estimated using separation factors (SF) determined as a ratio of distribution ratios of two metal ions:

SF = *D*_1_/*D*_2_. (11)

### 3.3. Nuclear Magnetic Resonance (NMR) Analysis

The samples for NMR analysis were prepared in CDCl_3_ and in the ionic liquid [C_1_C_4_im^+^][Tf_2_N^−^]. The concentration of 4-benzoyl-3-methyl-1-phenyl-2-pyrazolin-5-one (HL) was 1 × 10⁻^2^ mol/dm^3^, whereas that of the heterocyclic amines S1/S2 was 4 × 10⁻^3^ mol/dm^3^. The initial concentration of the investigated Eu^3+^ ion was 1 × 10^−3^ mol/dm^3^ (1:1 *v*/*v* extraction). All samples were prepared in 5 mm NMR tubes (type 507-PP-7) purchased from Rototec-Spintec GmbH (Bad Wildbad, Germany). The NMR analysis was conducted on a Bruker Avance NEO spectrometer (Bruker BioSpin, Ettlingen, Germany) operating at 600.18 MHz and at a temperature of 298.0 ± 0.1 K. The spectrometer was equipped with a nitrogen-cooled Prodigy probehead, and the chemical shifts were referenced to the diluent signal (7.26 ppm for CDCl_3_ and 7.41 ppm for H-5 of the ionic liquid). One-dimensional (1D) proton NMR experiments were conducted using the standard zg30 pulse sequence. The diluent signal (7.26 ppm for CDCl_3_ and 7.41 ppm for H-5 of the ionic liquid) was used as the reference. The NMR spectra of the ionic liquid solutions were acquired without lock. The unambiguous assignment of the majority of signals was achieved through the utilization of two-dimensional homonuclear and heteronuclear NMR experiments. In fact, in order to identify the signals that overlapped with the strong signals of the ionic liquid, a 1D TOCSY (total correlation spectroscopy) experiments were conducted. All data were processed manually using TopSpin 4.4.0 (Bruker BioSpin, Ettlingen, Germany). Zero-filling was employed to augment the number of data points, thereby enhancing the digital resolution. A window function with a 0.3 Hz width was applied prior to Fourier transformation in order to enhance the signal-to-noise ratio (S/N). Fourier transformation was employed to convert the time-domain data to frequency-domain NMR spectra. Zero- and first-order phase correction were meticulously adjusted to eliminate phase errors in the NMR spectra. Baseline correction was applied to ensure a flat baseline for accurate integration and peak analysis. Integration was performed using the Topspin 4.4.0 software integration tools to quantify the areas of the non-overlapping peaks.

## 4. Conclusions

In this study, the data on the solvent extraction of rare earth elements from aqueous nitrate solutions with synergistic mixtures of chelating ligand HL in combination with 1,10-phenanthroline or 2,2′-bipyridine in an ionic liquid were obtained. As outlined in detail, very slight enhancements of solvent extraction efficiencies were obtained in ionic media when utilizing the basic synergistic ligands, namely 1,10-phenanthroline and 2,2′-bipyridine. An effort was made to explore and understand the equilibrium mechanism, with the main goal of elucidating the nature of the mixed complexes formed in the organic phase. From the experimental results obtained through the slope analysis method and NMR spectroscopy, a model of the synergistic extraction mechanism was proposed. The ^1^H NMR spectroscopic analyses of the organic ionic liquid phases were performed to confirm the reaction mechanism of the extracted species with the use of synergistic reagents. This was performed to propose a definitive explanation for metal ion transfer into the IL. In addition, very weak interaction between the two extracting molecules (HL–1,10-phen or HL–2,2′-bipy) was also detected in the organic phase. However, this observation should not cause any influence on the synergistic process. It is anticipated that the findings of this investigation will prove beneficial in the design of synergistic solvent extraction systems for environmentally friendly and sustainable processes, thereby extending the applicability of bidentate nitrogen molecules and ILs in this field.

## Figures and Tables

**Table 1:** Values of the synergistic coefficients ([HPBI = 2.5 × 10^−3^ mol/dm^3^, [S] = 5 × 10^−3^ mol/dm^3^ phen: pH = 1.70; bipy: pH = 1.90) ([HTTA] = 4 × 10^−2^ mol/dm^3^ and [S] = 5 × 10^−3^ mol/dm^3^, phen: pH = 2.50; bipy: pH = 3.20) in CHCl_3_ [37].

Ln^3+^				logSCs		
	HL–Bipy	HL–Phen	HPBI–Bipy	HPBI–Phen	HTTA–Bipy	HTTA–Phen
La	0.57	0.09	1.41	3.26	2.91	4.82
Nd			2.48	3.58	4.11	6.04
Eu	−0.4	0.46	2.44	3.31	4.15	5.95
Ho			2.59	3.54	4.51	6.34
Lu			2.55	3.48	4.89	6.49

**Table 2:** Selectivity in 4f series during competitive solvent extraction.

System	Eu/La	Eu/Ce	Eu/Lu	Lu/La	Lu/Ce	Lu/Eu
IL	−	−	−	−	−	−
HL	15	7.1	0.8	17.5	8.2	1.2
S1	1.4	0.7	0.7	1.8	0.9	1.3
S2	−	−	−	−	−	−
HL–S1	19.2	10.6	0.8	23.6	13.1	1.2
HL–S2	12.5	8.9	0.8	14.7	10.5	1.2

**Table 3:** Separation factors of the pairs Eu/La and Lu/Eu, applying various molecular synergistic mixtures.

Extractants	Eu/La	Lu/Eu
HTTA–1,10-phen	3.3 × 10^3^	11.74
HTTA–2,2′-bipy	4.2 × 10^3^	18.62
HPBI–2,2′-bipy	263.02	5.62
HPBI–1,10-phen	27.54	6.45
HL/CHCl_3_	263	3.4
HL–diphenylsulphoxide [47]	75	13
HL–para-*tert*-octylcalix[4]arene with P=O donor groups [48]	213	5.6
HL–para-*tert*-butylcalix[4]arene with P=O donor groups [49]	60.2	12.6
HL–DB18C6–CHCl_3_ [50]	251.3	54.9
HL–B18C6 [50]	912	87
HL–18C6 [50]	371.5	147.9
HL–DB18C6–C_2_H_4_Cl_2_ [51]	72.4	251
HL–DB24C8 [51]	154.8	37.2
LIX54–1,10-phen [17]	2.75 × 10^3^	2.95
LIX54–2,2′-bipy [17]	1.6 × 10^4^	15.84

## Data Availability

Data are contained within the article.

## Supplementary Materials

The following supporting information can be downloaded at: https://www.mdpi.com/article/10.3390/molecules30040786/s1, Figure S1: Log *D*_L,S_ vs. log pH ([HL] = 5 × 10^−3^ mol/dm^3^ and [S] = 3 × 10^−3^ mol/dm^3^), log *D*_L,S_ vs. log[HL]_IL_ ([S] = 3 × 10^−3^ mol/dm^3^ at pH = 4.80) and log *D*_L,S_ vs. log[S] ([HL] = 5 × 10^−3^ mol/dm^3^ at pH = 2.85) plots for solvent extraction of Lu^3+^ with HP–bipy mixture in [C_1_C_4_im^+^][Tf_2_N^−^]. Figure S2: Log *D*_L,S_ vs. log pH ([HL] = 5 × 10^−3^ mol/dm^3^ and [S] = 3 × 10^−3^ mol/dm^3^), log *D*_L,S_ vs. log[HL]_IL_ ([S] = 3 × 10^−3^ mol/dm^3^ at pH = 3.60) plots for solvent extraction of Lu^3+^ with HP–phen mixture in [C_1_C_4_im^+^][Tf_2_N^−^]. Table S1: Chemical shifts (≤±0.01 ppm) of the compounds measured in CDCl_3_, in an IL and for the corresponding Eu-complexes in an IL at 298.0 ± 0.1 K. Figure S3: Representative NMR spectra of compounds 4, 7 and 10, respectively. Figure S4: NMR spectra of compounds 4, 7, and 10 in the region 7.1–9.4 ppm (100-fold amplified). Figure S5: Structural formulas of 4-benzoyl-3-methyl-1-phenyl-2-pyrazolin-5-one (HL), 1,10-phenantroline (S1), 2,2′-bipyridine (S2) and 1-butyl-3-methylimidazolium bis(trifluoromethylsulfonyl)imide [C_1_C_4_im^+^][Tf_2_N^−^].
