# Supplementary material for: Synergistic Solvent Extraction of Lanthanoids with Traditional Ligands (4-Acylpyrazolone and Bidentate Nitrogen Bases) in a Nontraditional Diluent Confirmed by Slope Analysis and NMR
Source: Molecules. 2025 Feb 8;30(4):786. doi: 10.3390/molecules30040786 (PMC11857860; doi:10.3390/molecules30040786)
Supplement: Supplementary file 1 [file molecules-30-00786-s001.zip › molecules-3444456-supplementary.pdf]

SUPPLEMENTARY MATERIAL

Synergistic Solvent Extraction of Lanthanoids with Traditional Ligands (4-Acylpyrazolone and Bidentate Nitrogen Bases) in a Nontraditional Diluent Confirmed by Slope Analysis and NMR

Maria Atanassova [1,*], Nina Todorova [1] and Svetlana Simova [2]

[1]University of Chemical Technologies and Metallurgy,
Department of General and Inorganic Chemistry,
8 Kliment Okhridski blvd., 1756 Sofia, Bulgaria
E-mail: ma@uctm.edu
[2]Institute of Organic Chemistry with Centre of Phytochemistry,
Bulgarian Academy of Sciences, Acad. G. Bonchev street, Block 9, 1113, Sofia, Bulgaria

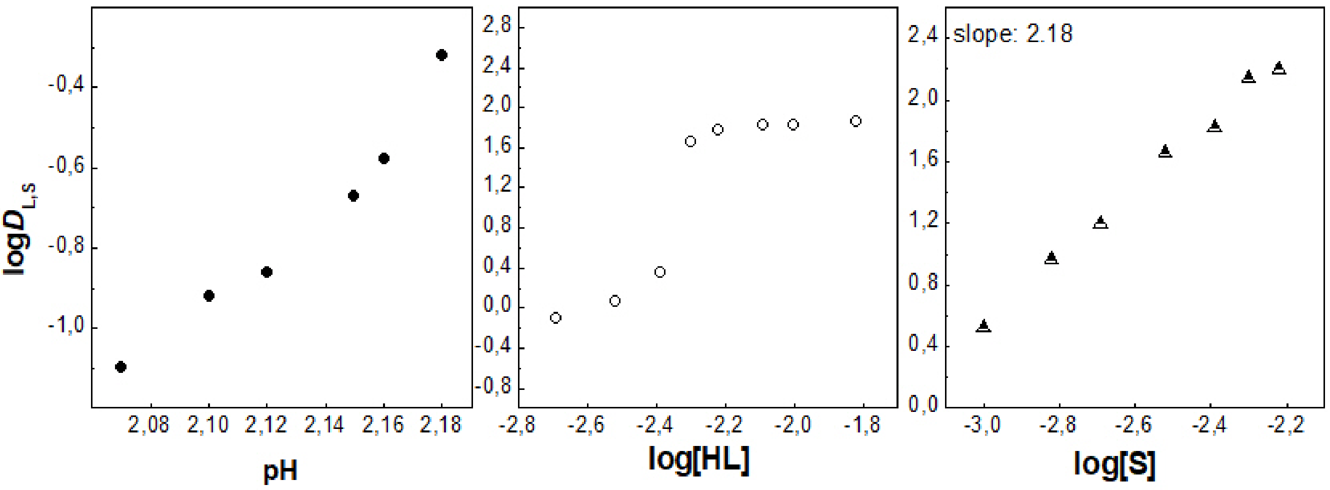

Figure S1. Log $D_{L,S}$ *vs*. log pH ([HL]=5×10^{-3} mol/dm^3 and [S]=3×10^{-3} mol/dm^3), log $D_{L,S}$ *vs*. log[HL]$_{IL}$ ([S]= 3×10^{-3} mol/dm^3 at pH=4.80) and log $D_{L,S}$ *vs*. log[S] ([HL]= 5×10^{-3} mol/dm^3 at pH=2.85) plots for solvent extraction of Lu^{3+} with HP–bipy mixture in [$C_1C_4im^+$][Tf_2N^-].

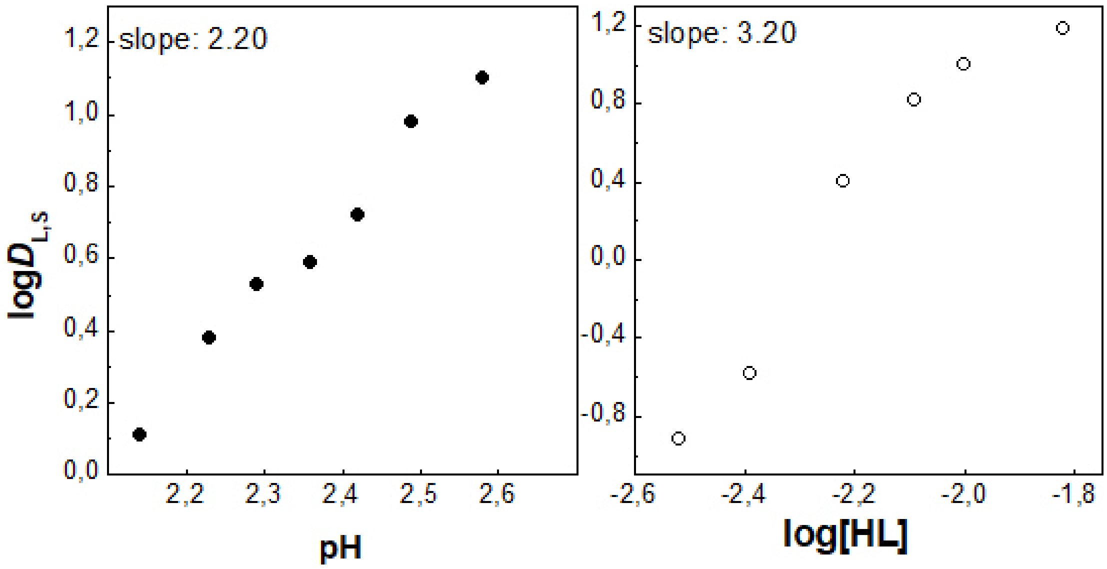

Figure S2. Log $D_{L,S}$ *vs*. log pH ([HL]=5×10^{-3} mol/dm^3 and [S]=3×10^{-3} mol/dm^3), log $D_{L,S}$ *vs*. log[HL]$_{IL}$ ([S]= 3×10^{-3} mol/dm^3 at pH=3.60) plots for solvent extraction of Lu^{3+} with HP–phen mixture in [$C_1C_4im^+$][Tf_2N^-].

Table S1. Chemical shifts (≤ ±0.01 ppm) of the compounds measured in $CDCl_3$, in an IL and for the corresponding Eu-complexes in an IL at 298.0±0.1K.

Compounds<br>Proton No.	**1**	**2**	**3**	**4**	**5**	**6**	**7**	**8**	**9**	**10**	**11**
**HL**											
H-2’	7.88			7.80			7.80	7.81	7.77	7.77	7.78
H-3’	7.48			7.48			7.46	7.46	7.46	7.46	7.47
H-4’	7.31			7.32			7.29	7.29	7.30	7.30	7.30
H-2”	7.64			7.69			7.67	7.68	7.68	7.68	7.68
H-3”	7.51			7.54			7.52	7.52	7.53	7.52	7.52
H-4”	7.58			7.60			7.57	7.58	7.59	7.58	7.58
H-8	2.10			2.07			2.10	2.10	2.10	2.10	2.11
**S1/S2**											
H-2		9.20	8.69		9.12	8.66	9.12	8.66		9.28	8.81
H-3		7.65	7.32		7.76	7.39	7.79	7.39		8.14	7.75
H-4		8.26	7.83		8.49	7.91	8.53	7.91		8.96	8.25
H-5			8.41			8.33		8.33			8.50
H-6		7.80			7.96		7.98			8.25	
$[C_1C_4im^+][Tf_2N^-]$	H-2	H-4	H-5	H-6	H-1’	H-2’	H-3’	H-4’			
	8.59	7.49	7.41	3.91	4.18	1.85	1.34	0.90			

Compounds:

1. 4-benzoyl-3-methyl-1-phenyl-2-pyrazolin-5-one (**HL**) in $CDCl_3$

2. 1,10-phenantroline (**S1**) in $CDCl_3$

3. 2,2’-bipyridine (**S2**) in $CDCl_3$

4. **HL** in $[C_1C_4im^+][Tf_2N^-]$

5. **S1** in $[C_1C_4im^+][Tf_2N^-]$

6. **S2** in $[C_1C_4im^+][Tf_2N^-]$

7. HL + **S1** in $[C_1C_4im^+][Tf_2N^-]$

8. HL + **S2** in $[C_1C_4im^+][Tf_2N^-]$

9. Eu^{3+} complex of **HL** in $[C_1C_4im^+][Tf_2N^-]$

10. Eu^{3+} complex of **HL/S1** in $[C_1C_4im^+][Tf_2N^-]$

11. Eu^{3+} complex of **HL/S2** in $[C_1C_4im^+][Tf_2N^-]$

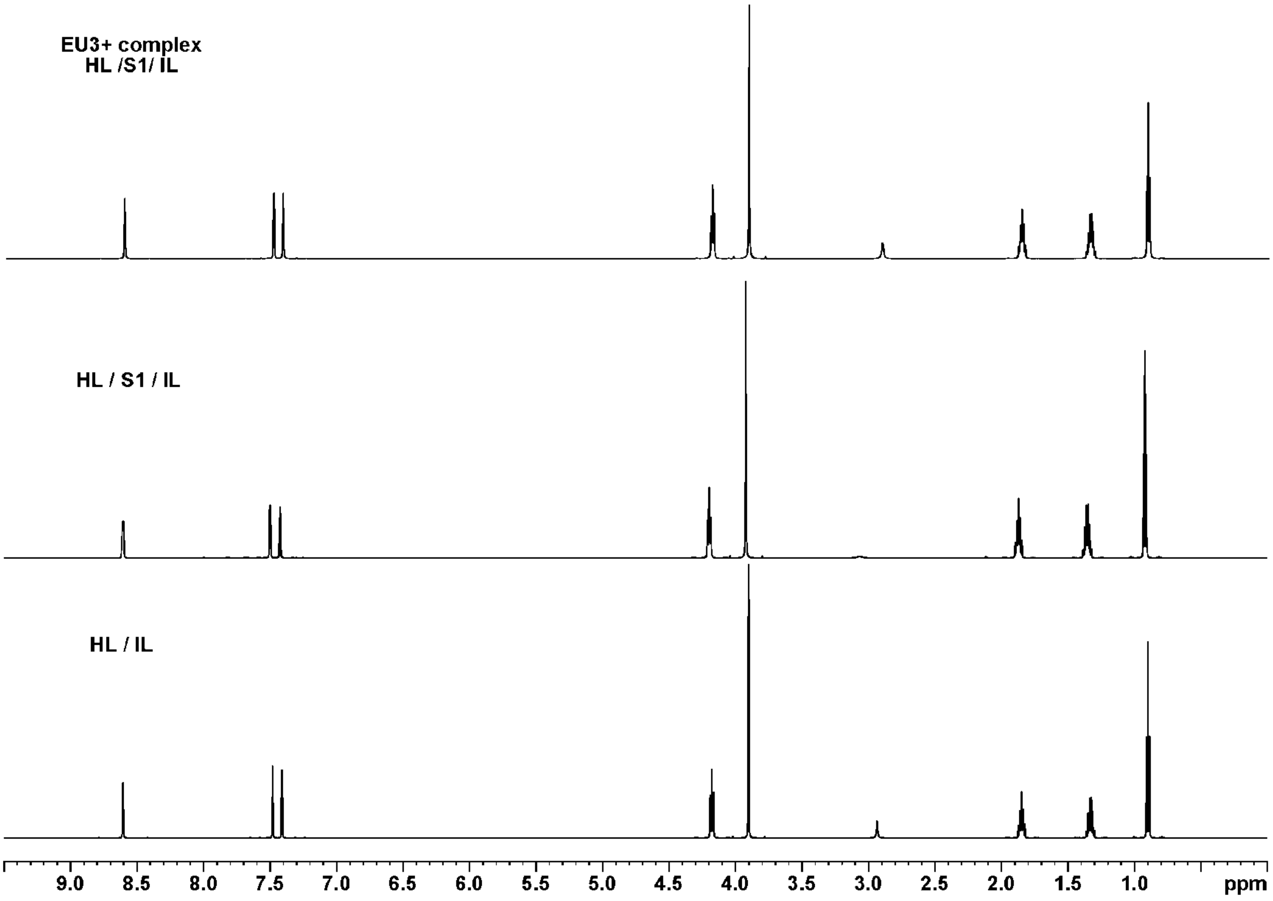

Figure S3. Representative NMR spectra of compounds 4, 7 and 10, respectively.

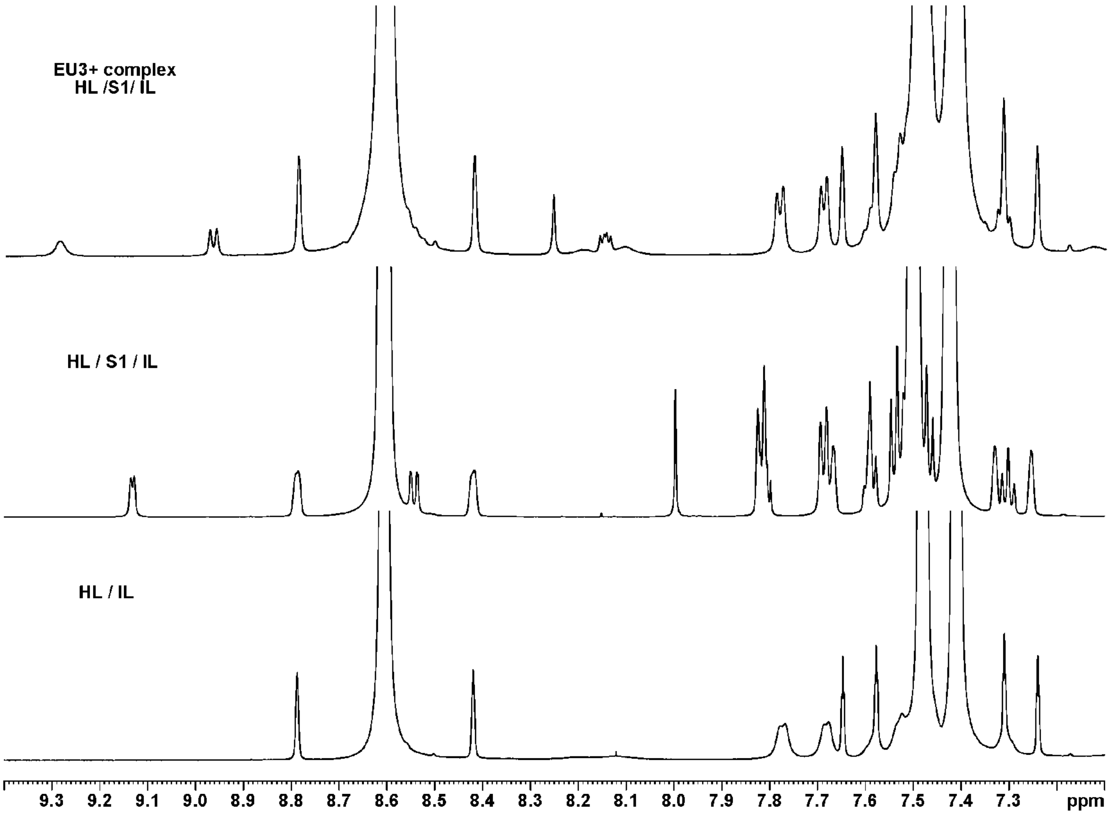

Figure S4. NMR spectra of compounds 4, 7, and 10 in the region 7.1-9.4 ppm (100-fold amplified).

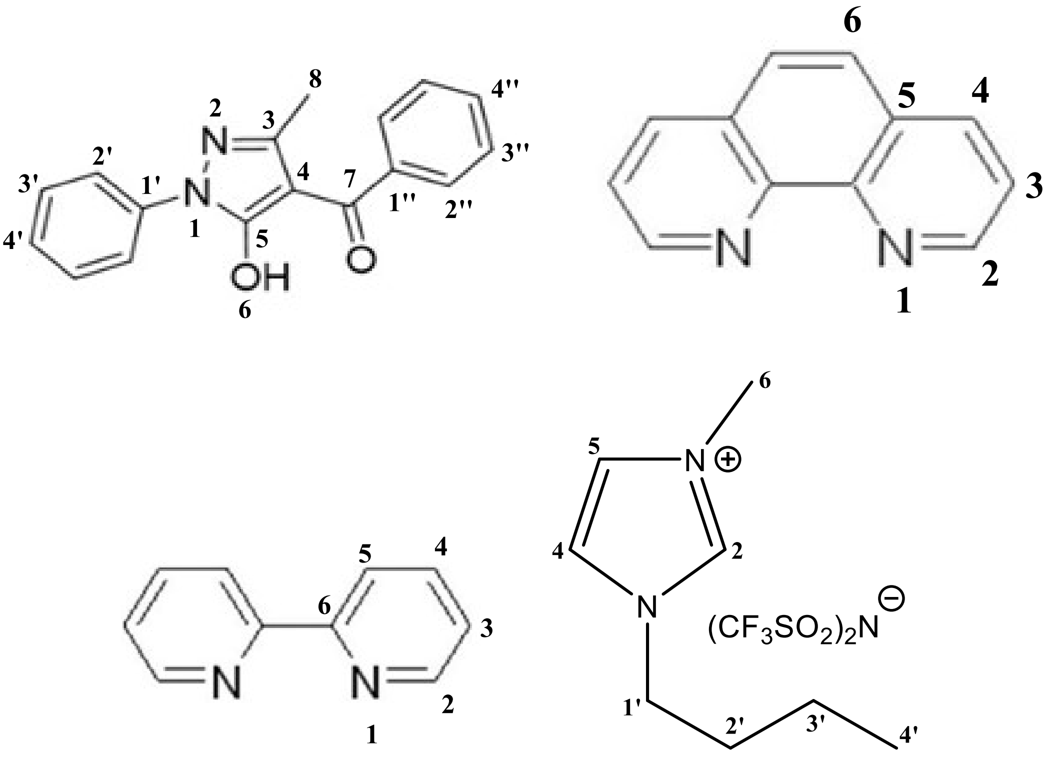

Figure S5. Structural formulas of 4-benzoyl-3-methyl-1-phenyl-2-pyrazolin-5-one (HL), 1,10-phenantroline (S1), 2,2’-bipyridine (S2) and 1-butyl-3-methylimidazolium bis(trifluoromethylsulfonyl)imide $[C_1C_4im^+][Tf_2N^-]$.
